# Practical observations - the COVID-19 influence on latvian early intervention work with first episode psychosis (FEP) patients

**DOI:** 10.1192/j.eurpsy.2021.793

**Published:** 2021-08-13

**Authors:** N. Kokina, L. Sile, B. Butorins, A. Pocopko, A. Lesina, S. Kikuste, I. Sapele, E. Rancans

**Affiliations:** 1 Department Of Mental Health Care, Daugavpils Psychoneurological Hospital, Daugavpils, Latvia; 2 Department Of Doctoral Studies, Riga Stradins University, Riga, Latvia; 3 Department Of Psychiatry And Narcology, Riga Stradins University, Riga, Latvia

**Keywords:** intervention, COVID-19, psychosis, rehospitalization

## Abstract

**Introduction:**

COVID-19 is a very stressful experience for people with FEP and changed the work routine of the mental health services they have used.

**Objectives:**

In this work, we aim to explore how the restrictions influenced the out-patient visits and rehospitalization rates.

**Methods:**

The Latvian Early intervention programme (Berze et al.,2019) for patients with FEP had started on 1^st^ January, 2019. The 1^st^ group of patients (n=28) finished the programme on 31^st^ December, 2019. The 2nd group of patients were enrolled in programme on 1^st^ January, 2020 (n=12). When on 12th of March in Latvia health care restriction started due the COVID-19 situation, we were forced to change our structure of LAT-EIP.

**Results:**

The average patient age of patients was 29.9 (SD±7.1) years in the 1^st^ group, in 2^nd^ group average age is 26.2 (SD±5.9) accordingly in years. In the 1^st^ group 60.7% of patients visited the psychiatrist 6-10 times vs 8.33% in 2^nd^ group, in the 1^st^ group 32.1 % of patients had 2-5 visits with psychiatrist vs 75% in 2^nd^ group. There were 72 family sessions in 1^st^ group, whereas in 2^nd^ group the family sessions were excluded. During the programme 7% (n=2) of patients in the 1^st^ group were rehospitalized vs 25% (n=3).

**Conclusions:**

The structure of LAT-EIP had change at the time of COVID-19 restrictions, unfortunately our work lack the statistical power. From the descriptive statistics, we can speculate that the rehospitalization rate is higher because of the lack of regular contact with psychiatrist and the lack of psychoeducation with families.

